# What Has Become of the Metric System?

**Published:** 1881-02

**Authors:** 


### WHAT HAS BECOME OF THE METRIC SYSTEM?

Less than a year ago the Metric System set the world ablaze and threatened annihilation to every man who should decline adopting it. One of its advocates, writing in a leading Eastern journal, denounced the common method as "the systemless system of weights and measures 'of the forefathers,' which, supported by selfish *vis inertiæ* and indolent conservatism, is now rapidly half tottering, half sneaking 'down the back entry of time.'" Just now the sneaking appears to be on the other side. If the metric men do not bestir themselves they are likely to vanish, together with their system, down the back stairs of oblivion. Perhaps they would succeed better with less bullying. One hates to be goaded and kicked even to a good dinner.

